# A longitudinal neuroimaging dataset on language processing in children ages 5, 7, and 9 years old

**DOI:** 10.1038/s41597-021-01106-3

**Published:** 2022-01-10

**Authors:** Jin Wang, Marisa N. Lytle, Yael Weiss, Brianna L. Yamasaki, James R. Booth

**Affiliations:** 1 Department of Psychology and Human Development, Vanderbilt University, Nashville, TN 37212 USA; 2 Department of Psychology, The Pennsylvania State University, University Park, PA 16801 USA; 3 Institute for Learning & Brain Sciences, University of Washington, Seattle, WA 98195 USA

**Keywords:** Human behaviour, Neurology

## Abstract

This dataset examines language development with a longitudinal design and includes diffusion- and T1-weighted structural magnetic resonance imaging (MRI), task-based functional MRI (fMRI), and a battery of psycho-educational assessments and parental questionnaires. We collected data from 5.5-6.5-year-old children (ses-5) and followed them up when they were 7-8 years old (ses-7) and then again at 8.5-10 years old (ses-9). To increase the sample size at the older time points, another cohort of 7-8-year-old children (ses-7) were recruited and followed up when they were 8.5–10 years old (ses-9). In total, 322 children who completed at least one structural and functional scan were included. Children performed four fMRI tasks consisting of two word-level tasks examining phonological and semantic processing and two sentence-level tasks investigating semantic and syntactic processing. The MRI data is valuable for examining changes over time in interactive specialization due to the use of multiple imaging modalities and tasks in this longitudinal design. In addition, the extensive psycho-educational assessments and questionnaires provide opportunities to explore brain-behavior and brain-environment associations.

## Abstract (summary)

**Table:** 

Measurement(s)	brain activity measurement • behavior or behavioral disorder measurement • Institutional Review Board Independent Ethics Committee Subject Questionnaire • brain structure
Technology Type(s)	functional magnetic resonance imaging • standardized testings • Questionnaire • Magnetization-Prepared Rapid Gradient Echo MRI • Diffusion Tensor Imaging
Sample Characteristic – Organism	Homo sapiens

Machine-accessible metadata file describing the reported data: 10.6084/m9.figshare.16726393

## Background & Summary

The preschool to elementary school period is crucial for language and reading development. This phase is marked by a mastery of complex morpho-syntactic principles^1^, an elaboration and refinement of semantic representations^2^, as well as dramatic changes in children’s ability to process phonemic information^3,4^. This phase is also accompanied with rapid reading development^5^. Understanding brain changes underlying language and reading development in this fast-developing period is important for facilitating early language and reading acquisition and designing interventions for those who struggle. Neural data on language acquisition is also important for testing neuro-cognitive theories of brain development. According to the *Interactive Specialization* account by Johnson^6^, the cerebral cortex begins with broad functionality. Then, over the course of development, activation within select regions becomes focal or specialized, with greater specialization related to better skill. This specialization of the brain is both intrinsically self-organized and influenced by environmental stimulation. Although this account has been supported by studies in face processing, social cognition, reading, and cognitive control^6^, most previous developmental neuroimaging studies investigating language development have either only cross-sectionally examined children with wide age ranges^7,8^ or have not examined multiple linguistic tasks^9,10^. Therefore, how the brain specializes for language in developing children is not known. In addition, several developmental disorders have been characterized as having delayed or atypical patterns of specialization^6^. Thus, it is important to study the neural basis of language development in typically developing children, because only by understanding normative trajectories can we discover what is different in children with developmental language disorders. While developmental language disorders are prevalent^11^, we still do not understand how interactive specialization is affected in this population.

**Fig. 1:**
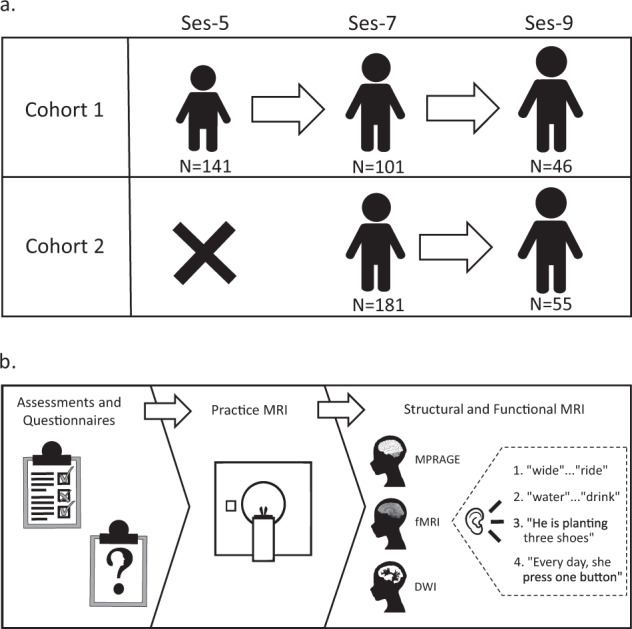
Overview of the study design. Illustration of (**a**) participant samples and (**b**) procedures at each session, which included assessments and questionnaires, practice magnetic resonance imaging (MRI), actual structural (MPRAGE), functional MRI (fMRI), diffusion-weighted imaging (DWI), and examples of stimuli from the functional MRI tasks.

This dataset examines the development of word- and sentence-level language processes in young 5-, 7-, and 9-year-old children using a longitudinal design with both neuroimaging and behavioral measurements. The functional magnetic resonance imaging (fMRI) tasks allow for an investigation of phonological, semantic, and syntactic processing. The T1- and diffusion-weighted imaging allow for an examination of brain structure. The standardized assessments provide opportunities for examining individual differences in oral language skills, reading ability, non-verbal intelligence, articulation quality and language variation status. The questionnaires assess potential behavior problems, attention deficit hyperactivity disorder symptoms, home environment, and parental attitude. Three hundred and twenty-two participants were included in this dataset. These participants completed at least one anatomical and one functional scan at one time point. Two cohorts of children were recruited for this project. The first cohort included 141 participants, who were enrolled when they were 5.5–6.5 years old (ses-5). Then, 101 out of the 141 were scanned approximately 1.5 years later when they were 7–8 years old (ses-7). After that, 46 out of the 101 were scanned again about 1.5 years later when they were 8.5–10 years old (ses-9). The second cohort included 181 participants, for whom their first visit was when they were 7–8 years old (ses-7). Then, 55 participants in the second cohort were followed up approximately 1.5 years later when they were 8.5–10 years old (ses-9). Figure 1 provides an overview of the study design and the number of participants recruited at each time point.

This dataset, with its longitudinal design, allows researchers to more effectively test theories of brain development. Having multiple time points within an individual will allow investigations into how early brain function and structure drives subsequent development. For example, one could test one of the central assumptions of the Interactive Specialization account, that early connectivity will determine later specialization^6^. Given that this dataset also has multiple imaging tasks for each individual, one will be able to directly test for developmental changes in specialization at the word- and sentence-level. Not only will the longitudinal data allow one to test theories of brain development, but it will also allow for testing predictive models that should be useful for early identification of children who show slow language and reading growth^12,13^. This has direct implications for prevention through early intervention. This dataset also provides extensive phenotype information in the form of standardized assessments and questionnaires, so, for example, it is a fertile testing bed for examining the relation between individual differences in language skill and environmental variables. One could also investigate, in line with the Interactive specialization account, that environmental factors shape brain development^6^.

Here we describe the public neuroimaging and behavioral dataset entitled “A longitudinal neuroimaging dataset on language processing in children ages 5, 7, and 9 years old” available on the OpenNeuro project (https://openneuro.org) and organized in compliance with the Brain Imaging Data Structure (BIDS). We hope that our sharing of the raw data will aid in the reproducibility^14^, reliability^14^, and impact^15,16^ of neuroimaging research.

## Methods

### Participants

The dataset includes 322 participants, recruited from the Austin, Texas metropolitan area via online advertisements (i.e., Facebook, lab webpage ads), community fliers, brochures sent to schools and clinics, and mailings distributed to parents. Advertisements and brochures focused on children with language impairment as well as typically developing children in an effort to recruit a diverse sample. A detailed description of the number of participants included in each task along with the distribution of participant sex is provided in Table 1. All experimental procedures were approved by the Institutional Review Board at The University of Texas at Austin. Written consent from parents and assent from participants were obtained. Participants were screened for eligibility via an exclusionary survey filled out by guardians. Exclusionary criteria for this project included participants who: (1) had any metal on/in the body that could not be removed (e.g., braces, implants, etc.), (2) were claustrophobic, (3) spent more than 40% of their time speaking a non-English language, (4) had a history of premature birth or birth complications, (5) had a hearing impairment or an uncorrected visual impairment, or (6) had attention deficit hyperactivity disorder (ADHD), a psychiatric disorder, or a neurological diagnosis.

**Table 1:** Number of participants for each scan at each time point.

Time Point	Scan Type/Name			Number of participants		
				Male	Female	Total
Ses-5	T1 weighted			55	86	141
	T2 weighed	Sound Task	Run-01	52	68	120
			Run-02	49	68	117
		Meaning Task	Run-01	36	55	91
			Run-02	36	53	89
		Plausibility Task	Run-01	33	50	83
			Run-02	34	49	83
		Grammaticality Task	Run-01	48	68	116
			Run-02	48	69	117
	Diffusion weighted			36	51	87
Ses-7	T1 weighted			131	151	282
	T2 weighed	Sound Task	Run-01	119	131	250
			Run-02	121	130	251
		Meaning Task	Run-01	99	117	216
			Run-02	100	116	216
		Plausibility Task	Run-01	98	116	214
			Run-02	97	116	213
		Grammaticality Task	Run-01	117	133	250
			Run-02	114	136	250
	Diffusion weighted			88	103	191
Ses-9	T1 weighted			43	57	100
	T2 weighed	Sound Task	Run-01	43	58	101
			Run-02	43	58	101
		Meaning Task	Run-01	37	49	86
			Run-02	37	50	87
		Plausibility Task	Run-01	39	51	90
			Run-02	39	50	89
		Grammaticality Task	Run-01	43	58	101
			Run-02	41	56	97
	Diffusion weighted			38	46	84

Participants having at least one functional imaging scan regardless of quality were included in this data sample. The number of participants for each time point and the distribution of participant sex are displayed.

### Psycho-educational Assessments and Questionnaires

Parents completed a series of questionnaires and participants completed a handedness interview as well as several standardized psycho-educational assessments at each time point to measure a variety of cognitive abilities. The questionnaires completed by parents included the exclusionary survey, exclusionary survey follow-up, developmental survey, Cognitive Stimulation survey^17^, Parent Knowledge of Child Language Development (PKCLD) survey^18^, the Conners Early Childhood survey^19^, the Conners Comprehensive Behavior Rating Scales^20^, and the Attention Deficit Hyperactivity Disorder (ADHD) Rating Scale–IV: Home Version^21^. Only a subset of questions from the exclusionary survey, the exclusionary survey follow-up, and the developmental survey are shared due to confidentiality issues. The non-shared questions included identifying information such as addresses, contact information, and the names of children’s schools or clinics. For the other questionnaires, all questions are shared. The questions in the Cognitive Stimulation survey were adapted from the first and third grade parent interview questions from the Early Childhood Longitudinal Studies (ECLS) program^17^. The PKCLD survey is a self-developed questionnaire on parent knowledge of child language development that was published in a previous study (Table 5 in that study)^18^. The exclusionary and developmental surveys were only filled out on participants’ first visit and the exclusionary follow-up survey was only filled out for subsequent visits. Children were asked to perform 5 actions to observe their handedness. Specifically, they were asked to write their name, erase a drawing, turn over a card, open a container and throw a ball. Table 2 shows the questionnaires completed at each time point.

**Table 2:** Questionnaires completed at each time point.

Name	Measure	Ses-5	Ses-7	Ses-9	Scores
Exclusionary survey	Eligibility screening on child’s non-English language usage and ADHD diagnosis status	*	*		None
Exclusionary survey follow-up	Eligibility re-screening on child’s non-English language usage, ADHD and other developmental disorder diagnosis status		*	*	None
Developmental survey	Children’s language history, daily activities, disorder evaluation/diagnosis, family history of disorders, and demographic information as well as parental education, income, and occupation	*	*		None
Cognitive Stimulation	Children’s learning environment and activities at home		*	*	None
Parent Knowledge of Child Language Development	Parent knowledge of child’s language development		*	*	None
Conners Early Childhood	Child behavior	*			RS TS
Conners Comprehensive Behavior Rating Scales	Child behavior		*	*	
ADHD Rating Scale-IV: Home Version	Hyperactivity and Impulsivity (HI)	*	*	*	RS Percentile
	Inattention (IA)	*	*	*	
	Total	*	*	*	
Handedness	Action with hands	*	*	*	

Note: None indicates that no scores were calculated; RS refers to Raw Score; TS refers to T Score.

Standardized assessments included the Clinical Evaluation of Language Fundamentals (CELF-5)^22^, the Comprehensive Test of Phonological Processing (CTOPP-2)^23^, the Woodcock-Johnson III Tests of Achievement (WJ-III)^24^, the Kaufman Brief Intelligence Test (KBIT-2)^25^, the Goldman Fristoe-2 Test of Articulation (GFTA-2)^26^, and the Diagnostic Evaluation of Language Variation (DELV)^27^. Standardized assessment administration always began with the CELF-5, and then continued with the other standardized tests. Online-Only Table 1 shows the names of the standardized assessments and their subtests completed at each time point as well as score types.

### Practice Imaging

All participants completed a practice MRI session in a mock scanner at least once prior to the first imaging session at each time point. The practice session allowed participants to become familiar with the in-scanner tasks as well as the scanning environment. The practice session was used to reduce participants’ anxiety when completing the real MRI, to train participants to remain still in the scanner, and to increase participants’ task understanding.

In each practice session, experimenters first explained the rules of the task and demonstrated with a few trials to make sure that the participant understood the tasks. Then, participants completed practice versions of each task in the mock scanner. Each practice task was the same as the in-scanner task in terms of trial type and timing. No word pairs or sentences used in the practice tasks were used in the functional imaging tasks. Behavioral performance was not collected for the practice sessions.

### Imaging Acquisition

All neuroimaging data were collected using a Siemens Skyra 3 T MRI scanner located at The University of Texas at Austin Imaging Research Center. All images were acquired using a 64-channel head coil. Participants were positioned supine in the MRI scanner and foam pads were placed around the head to minimize movement. Participants were given a right-hand response box to respond to the functional imaging tasks. Visual stimuli were projected on a screen behind the scanner which participants viewed via a mirror attached to the head coil. Audio stimuli were presented through sound attenuating earphones to minimize the effects of scanner noise. During structural MRI and diffusion weighted imaging, participants watched a movie to increase comfort. Participants were encouraged to remain still and were given breaks to talk to an experimenter between scans.

#### Structural MRI

T1-weighted Magnetization Prepared - RApid Gradient Echo (MPRAGE) images were collected using GRAPPA, a parallel imaging technique based on k-space, and the following parameters: GRAPPA accel.factor PE = 2, TR = 1900 ms, TE = 2.43 ms, field of view = 256 mm, matrix size = 256 × 256, bandwidth = 180 Hz/Px, slice thickness = 1 mm, number of slices = 192, voxel size = 1 mm isotropic, flip angle = 9°.

#### Functional MRI

Blood oxygen level dependent (BOLD) signal was acquired using a T2-weighted susceptibility weighted single-shot echo planar imaging (EPI) and the following parameters: TR = 1250 ms, TE = 30 ms, field of view = 256 mm, matrix size = 128 × 128, bandwidth = 1776 Hz/Px, slice thickness = 2 mm without a gap, number of slices = 56, voxel size = 2 mm isotropic, flip angle = 80°, multi-band acceleration factor = 4. Slices were acquired interleaved from foot-to-head.

#### Diffusion Weighted Imaging

Diffusion weighted images were collected using single-shot echo planar imaging (EPI) with the following parameters: GRAPPA accel.factor PE = 2, TR = 5000 ms, TE = 71 ms, field of view = 256 mm, matrix size = 128 × 128, bandwidth = 1502 Hz/Px, slice thickness = 2 mm, number of slices = 57, voxel size = 2 mm isotropic, flip angle = 90°, multi-band acceleration factor = 3, *b-value1* = 0 s/mm^2^, *b-value2* = 800 s/mm^2^, echo spacing = 0.75 ms, diff.directions = 64. Slices were acquired interleaved from foot-to-head.

#### Field Map

The field map acquisition was a 3D interleaved dual echo gradient echo pulse sequence, with the following parameters: TR = 600 ms, TE1 = 4.92 ms, TE2 = 7.38 ms, field of view = 192 mm, matrix size = 64 × 64, bandwidth = 300 Hz/Px, slice thickness = 3 mm, number of slices = 57, voxel size = 3 mm isotropic, flip angle = 60°. Slices were acquired interleaved from foot-to-head.

### Functional MRI tasks

**Fig. 2:**
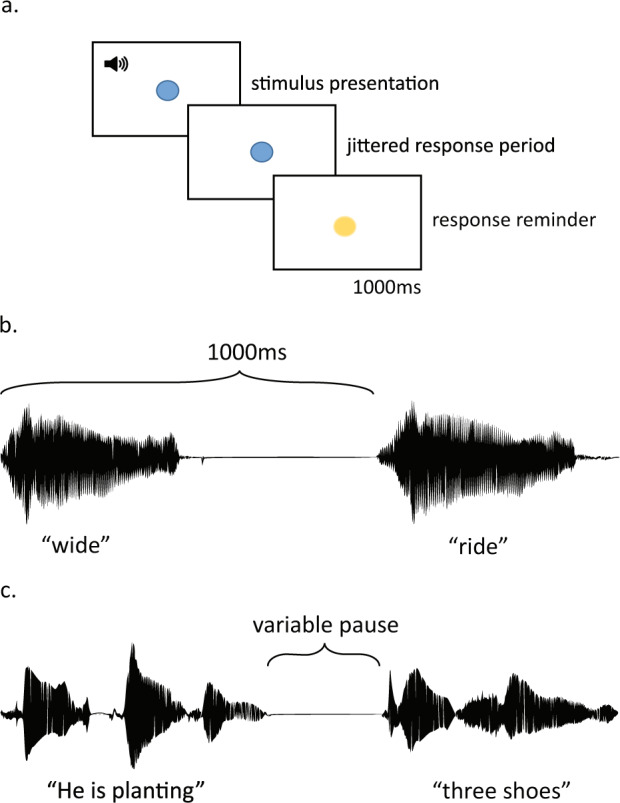
Functional MRI task and stimulus design. Illustration of (**a**) trial procedure used in all tasks, (**b**) soundwave for word-level stimuli including a pause inserted between the two words, and (**c**) soundwave for sentence-level stimuli including a variable pause inserted between phrases.

There were four in-scanner functional MRI tasks: a sound task, a meaning task, a plausibility task, and a grammaticality task. All four tasks followed a similar stimulus presentation procedure. Figure 2a shows the general procedure of a trial. Participants typically completed all four tasks in two scan days with two tasks (2 runs in each task) assigned for each scan day. The total scan duration for each day was approximately 1 hour. The diffusion weighted imaging and field map were acquired when there was extra time after children finished all of the functional MRI tasks on that scan day. Experimenters eyeballed the data quality in terms of movement during and/or after each scan and repeated the scans that they determined as bad if time allowed. Participants could be invited for scanning on additional days if they had not finished the four functional tasks.

### Sound Task

The sound task is an auditory phonological awareness task that taps into children’s phonological processing for spoken words. In this task, participants were sequentially presented with two auditory one-syllable words and were asked to judge whether the two words they heard shared any of the same sounds with a button box held in their right hand. The task included three different experimental conditions: rhyme (P_R), onset (P_O), and unrelated (P_U). Participants were expected to respond ‘yes’ with their right index finger for both the rhyme and onset conditions and to respond ‘no’ with their right middle finger for the unrelated condition. In addition to the three experimental conditions, the task included a perceptual/motor control (P_C) condition in which participants heard two sequentially presented frequency modulated sounds and were expected to press the ‘yes’ button whenever they heard the “shh-shh” sound. Table 3 shows example stimuli from each condition. The task included a total of 96 trials divided into two separate 48-trial runs with each run containing 12 trials per condition. Within a trial, each auditory word stimulus had a duration ranging from 439 to 706 ms. The second word was presented approximately 1000 ms after the onset of the first one. Overall, in each trial, the total stimuli duration (i.e., two words with a brief pause in between) ranged from 1490 to 1865 ms and was followed by a jittered response interval that varied between 1500 and 2736 ms. A blue circle appeared simultaneously with the auditory presentation of the stimuli to help maintain attention on the task. The blue circle changed to yellow to provide a 1000 ms warning for the participants to respond, if they had not already, before moving on to the next trial. The total trial duration ranged from 3000 to 4530 ms. Each run lasted approximately 3 min.

**Table 3:** Stimulus examples for each condition in each imaging task.

Task	Condition	Example	Description	Expected Response
**Word Level Tasks**				
Sound Task	Rhyme (P_R)	Wide-Ride	Two words share the final sound (i.e., rhyme)	Yes
	Onset (P_O)	Coat-Cup	Two words share the first sound	Yes
	Unrelated (P_U)	Zip-Cone	Two words do not share sounds	No
	Perceptual Control (P_C)	Shh-Shh	Frequency modulated noise	Yes
Meaning Task	Low Association (S_L)	Water-Drink	Two words have a weak semantic association	Yes
	High Association (S_H)	Syrup-Pancake	Two words have a strong semantic association	Yes
	Unrelated (S_U)	Flush-Cliff	Two words have no semantic association	No
	Perceptual Control (S_C)	Shh-Shh	Frequency modulated noise	Yes
**Sentence Level Tasks**				
Plausibility Task	Strongly Congruent (SP_S)	She is singing one song.	The verb and the object have a strong association	Yes
	Weakly Congruent (SP_W)	She does not taste two foods.	The verb and the object have a weak association	Yes
	Incongruent (SP_I)	He is planting three shoes.	The verb and the object share no semantic association	No
	Perceptual Control (SP_C)	Shh-Shh	Frequency modulated noise	Yes
Grammaticality Task	Finiteness Violation (G_F)	Every day, she press one button.	The sentence has a grammatical violation on the verb form.	No
	Grammatically Correct (G_G)	She is moving one box.	The sentence has no grammatical violation.	Yes
	Plurality Violation (G_P)	Every day, they stir three pot.	The sentence has a grammatical violation in number and object agreement.	No
	Perceptual Control (G_C)	Shh-Shh	Frequency modulated noise	Yes

All individual words were recorded using Audacity (https://www.audacityteam.org/) in one long stream with pauses in between by a female native English speaker in a sound-insulated booth to ensure all the words were recorded in the same environment. To make the stimulus files for our experiment, the recording was first segmented into single words, and then word pairs were combined with a brief pause in between to ensure the onset of the second word was 1000 ms after the onset of the first word (see Fig. 2b) using Praat (http://www.fon.hum.uva.nl/praat/). The three experimental conditions were designed according to the following standards. The rhyme (P_R) condition was defined as word pairs sharing the same vowel and final phoneme/cluster (corresponding to 2-3 letters at the end of their written form). The onset (P_O) condition was defined as word pairs sharing the same initial phoneme (corresponding to 1 letter of their written form). The unrelated (P_U) condition was defined as word pairs that had no shared phonemes (and no letters in their written form). All words were monosyllabic with only one morpheme. There were no significant differences between conditions in word length, number of phonemes, written word frequency, orthographic neighbors, phonological neighbors, or semantic neighbors for either the first or the second word in a trial within a run (Rhyme vs. Onset: *p*s > 0.123; Rhyme or Onset vs. Unrelated: *p*s > 0.123) or across runs (Rhyme: *p*s > 0.162; Onset: *p*s > 0.436; Unrelated: *p*s > 0.436; linguistic characteristics were obtained from the English Lexicon Project https://elexicon.wustl.edu/)^28^. There were also no significant differences between conditions in phoneme probabilities for either the first or the second word in a trial either within a run (Rhyme vs. Onset: *p*s > 0.302; Rhyme or Onset vs. Unrelated: *p*s > 0.203) or across runs (Rhyme: *p*s > 0.097; Onset: *p*s > 0.313; Unrelated: *p*s > 0.542; phoneme probabilities were obtained from a phonotactic probability calculator https://calculator.ku.edu/phonotactic/English/words)^29^. In addition, orthographic and phonological consistency did not differ across conditions either within a run (*p*s > 0.053) or across runs (*p*s > 0.213). The orthographic and phonological consistency were computed using the 2998 mono-syllable words database^30^. In line with Bolger *et al*.^31^, phonological enemies were defined as the number of words with similar spelling, but different pronunciation of the rhyme and orthographic enemies were defined as the number of words with similar pronunciation, but different spelling of the rime. Friends were defined as the number of words with the same rime spelling and same rhyme pronunciation as the stimulus. The orthographic or phonological consistency was the ratio of friends to the sum of friends and enemies [friends/(friends + enemies)]. Words that have a ratio approaching 1.0 have a high orthographic or phonological consistency. In addition, all word pairs had no semantic association based on the University of South Florida Free Association Norms (http://w3.usf.edu/FreeAssociation/)^32^.

### Meaning Task

The meaning task examines children’s semantic processing for spoken words. In this task, participants were sequentially presented with two auditory words and were asked to indicate whether the two words they heard went together semantically using the button box held in their right hand. The task included three different experimental conditions: low association (S_L), high association (S_H), and unrelated (S_U) conditions. Participants were expected to respond ‘yes’ with their right index finger for both the low and high association conditions and to respond ‘no’ with their right middle finger for the unrelated condition. In addition to the three experimental conditions, the task included a perceptual/motor control (S_C) condition in which participants heard two sequentially presented frequency modulated sounds and were asked to press the ‘yes’ button whenever they heard the “shh-shh” sound. Table 3 shows example stimuli from each condition. The task included a total of 96 trials divided into two separate 48-trial runs, with 12 trials per condition per run. Within each trial, each auditory word had a duration ranging from 500 to 700 ms. The second word was presented approximately 1000 ms after the onset of the first one. Overall, in each trial, the stimuli duration (i.e., two words with a brief pause in between) ranged from 1500 to 1865 ms and was followed by a jittered response interval between 1800 and 2701 ms. A blue circle appeared simultaneously with the auditory presentation of the stimuli to help maintain attention on the task. The blue circle changed to yellow to provide a 1000 ms warning for the participants to respond, if they had not already, before moving on to the next trial. The total trial duration ranged from 3300 to 4565 ms. Each run lasted approximately 3 min.

All of the individual words in the meaning task were recorded and edited in the same way as in the sound task using Audacity and Praat. The three experimental conditions were designed according to the following standards. The low association (S_L) condition was defined as word pairs having a weak semantic association between 0.14 and 0.39 (M = 0.27, SD = 0.07). The high association (S_H) condition was defined as word pairs having a strong semantic association between 0.40 and 0.85 (M = 0.64, SD = 0.13). The unrelated (S_U) condition was defined as word pairs that shared no semantic association. Associative strength was derived from the Forward Cue-to-Target Strength (FSG) values from the University of South Florida Free Association Norms (http://w3.usf.edu/FreeAssociation/)^32^. There was no difference of association strength between the two runs of the meaning task (*p*s > 0.425). There were also no significant differences between conditions in word length, number of phonemes, number of syllables, written word frequency, orthographic neighbors, phonological neighbors, semantic neighbors, or number of morphemes for either the first or the second words in a trial either within a run (High vs. Low: *p*s > 0.167; High or Low vs. Unrelated: *p*s > 0.068) or across runs (High: *p*s > 0.069; Low: *p*s > 0.181; Unrelated: *p*s > 0.097; linguistic characteristics were obtained from the English Lexicon Project https://elexicon.wustl.edu/)^28^.

### Plausibility Task

The plausibility task examines children’s semantic processing skill at the sentence level. In this task, participants were presented with one auditory sentence per trial and were asked to judge whether the sentence they heard made sense. The task included three different experimental conditions: strongly congruent (SP_S), weakly congruent (SP_W), and incongruent (SP_I). Participants were expected to press the ‘yes’ button with their right index finger for both the strongly and weakly congruent conditions and press the ‘no’ button with their right middle finger for the incongruent condition. In addition to the three experimental conditions, the task included a perceptual/motor control condition (SP_C) in which participants heard frequency modulated noise and were asked to press the ‘yes’ button whenever they heard the “shh-shh” sound. Table 3 shows example stimuli from each condition. The task included a total of 80 trials divided into two separate 40-trial runs with 10 trials per condition per run for a total of 20 sentences for each of the four conditions. Stimuli duration ranged from 2748 to 4520 ms and was followed by a jittered response interval between 1875 and 3450 ms. A blue circle appeared simultaneously with the auditory presentation of the stimuli, to help maintain attention on the task. The blue circle changed to yellow to provide a 1000 ms warning for the participants to respond, if they had not already, before moving on to the next trial. The total trial duration ranged from 4694 to 7900 ms. Each run lasted approximately 4.5 min.

The auditory sentences were recorded in a sound-insulated booth using Audacity. All sentences were read by a female native English speaker with a natural speed, and a brief pause between phrases. Each auditory sentence had the following structure: an optional carrier phrase (e.g., “Last week”/ “Every day”) + subject and verb phrase (e.g., “she baked”) + number and object (e.g., “two cakes”). All recorded sentences were segmented by phrases and consistent pauses ranging from 483 to 643 ms were then added in between phrases using Praat, so that all sentences were similar in their pacing (see Fig. 2c). The sentences included one of the following four verb forms: (1) third-person present tense (-s); (2) present progressive copula (be); (3) auxiliary verb (do); or (4) simple past tense (-ed). The subject used (he/she/they), the verb form used, the number used (one/two/three), and the frequency of “not” usage in the sentences were approximately matched across runs. The three sentence conditions in the plausibility task were designed according to the following standards. The strongly congruent (SP_C) condition was defined as a high semantic association between the verb and the object in the sentence with an association strength between 0.28 and 0.81 (M = 0.41, SD = 0.12), whereas the weakly congruent (SP_W) condition was defined as a low semantic association between the verb and the object in the sentence with an association strength between 0.02 and 0.19 (M = 0.11, SD = 0.05). In the incongruent (SP_I) condition, the verb and the object in the sentence did not share semantic association. The association strengths were based on the Forward Cue-to-Target Strength (FSG) values from the University of South Florida Free Association Norms (http://w3.usf.edu/FreeAssociation/)^32^. There were no significant differences across all conditions within or across runs in sentence length (*p*s > 0.902). There were also no significant differences in averaged word frequency, averaged word length, or number of syllables across all conditions within a run (Strong vs. Weak: *p*s > 0.241; Strong or Weak vs. Incongruent: *p*s > 0.077) or across runs (Strong: *p*s > 0.136; Weak: *p*s > 0.547; Incongruent: *p*s > 0.473, linguistic characteristics were drawn from the English Lexicon Project https://elexicon.wustl.edu/)^28^.

### Grammaticality Task

The grammaticality task examines children’s syntactic processing skill. In this task, participants were presented with one auditory sentence per trial and were asked to judge whether the sentence they heard sounded right. The task included three different experimental conditions: finiteness violation (G_F), grammatically correct (G_G), and plurality violation (G_P). Participants were expected to press the ‘yes’ button with their right index finger to the grammatically correct condition and to press the ‘no’ button with their right middle finger to both the finiteness and plurality violation conditions. In addition, the task included a perceptual/motor control condition (G_C) in which participants heard frequency modulated noise and were asked to press the ‘yes’ button whenever they heard the “shh-shh” sound. Table 3 shows example stimuli from each condition. The task included a total of 80 trials divided into two separate 40-trial runs, with 10 trials per condition per run for a total of 20 sentences for each of the four conditions. In each trial, stimulus duration ranged from 2740 to 4972 ms and was followed by a jittered response interval between 1234 and 4611 ms. A blue circle appeared simultaneously with the auditory presentation of the stimuli, to help maintain attention on the task. The blue circle changed to yellow to provide a 1000 ms warning for the participants to respond, if they had not already, before moving on to the next trial. The total trial duration ranged from 5174 to 8422 ms. Each run lasted approximately 4.5 min.

The auditory sentences were recorded and structured in the same way as in the plausibility task. Consistent pauses ranging from 461 to 694 ms were added in between phrases to make sure that all sentences had similar pacing. The subject used (he/she/they), the verb form used, the number used (one/two/three/four/five/six), and the frequency of “not” usage in the sentences were approximately matched across runs. The three sentence conditions in the grammaticality task were designed according to the following standards. The plurality violation (G_P) condition was defined as a mismatch between the number and the object by either adding/omitting an “s” in the object noun word. The finiteness violation (G_F) condition was defined as an inconsistency between the subject and verb phrase by either adding/omitting an inflection or omitting/substituting an auxiliary verb. The two conditions with grammatical violations (i.e., G_F and G_P) were approximately matched in terms of the number of omissions versus addition/substitution across runs. The grammatically correct (G_G) condition was defined as sentences without grammatical errors. There were no significant differences across all conditions within or across runs in sentence length (*p*s > 0.489). There were also no significant differences in the averaged word frequency, averaged word length, or number of syllables across all conditions within a run (Finiteness vs. Plurality: *p*s > 0.172; Finiteness or Plurality vs. Grammatically correct: *p*s > 0.122) or across runs (Finiteness: *p*s > 0.306; Plurality: *p*s > 0.235; Grammatically correct: *p*s > 0.331, linguistic characteristics were drawn from the English Lexicon Project https://elexicon.wustl.edu/)^28^.

All tasks were presented using E-prime 2.0 (https://pstnet.com/products/e-prime). For all four tasks, the E-prime program included two windows per trial to collect accuracy and reaction time (RT). Window 1 started from the onset of each trial and lasted for a duration of 750 ms. Window 2 began at 750 ms and lasted until the end of the trial. The response for each trial was calculated from the critical onset of the current trial to the critical onset of the next trial. We defined the critical onset of a trial as the onset of a stimulus segment where participants could correctly make a judgment. For example, in the two word-level tasks, the critical onset for the experimental conditions was the onset of the second word (e.g., 1000 ms after the start of each trial). Participants could only correctly perform the task (i.e., judge whether the two words shared the same sounds or went together) after hearing the second word. In the plausibility task, the critical onset for the experimental conditions was the onset of the final phrase (i.e., number + object) in a sentence because participants could only correctly answer the question, whether the sentence they heard made sense or not, after hearing the end of the sentence. The same rule applied to the grammaticality task, except for the finiteness violation condition, in which participants could respond correctly when they heard the verb phrase (i.e., subject + verb) in a sentence. Thus, we defined the critical onset for the finiteness violation condition as the onset of the verb phrase in a sentence. The critical onset for the perceptual/motor control condition in all tasks was the onset of the trial. As described below, both calculated and raw response and RT are provided for all tasks. The approach taken to calculate accuracy and RT based on raw responses and RT from window 1 and window 2 can be traced using our shared code and calculation document, which is described in Section 4.

## Data Records

This dataset^33^ is made public under the Creative Commons CCO license and hosted on the OpenNeuro platform (https://openneuro.org/). The data is organized in accordance with the Brain Imaging Data Structure (BIDS) specification version 1.5.0 (https://bids-specification.readthedocs.io/en/stable/). BIDS is an organizational and naming convention for neuroimaging and behavioral data created to facilitate understanding and ease of use when sharing data. Openneuro.org provides a built-in BIDS validation tool that screens all uploaded datasets to ensure compliance with the BIDS specification. Any warnings generated by the BIDS validation tool for this dataset are explained in the known issues section of the readme.txt file included in the dataset.

All neuroimaging data is in the compressed Neuroimaging Informatics Technology Initiative (NIfTI) format (nii.gz). All tabular data files are in tab-separated values text file format (tsv) and all data dictionary descriptor files are in JavaScript Object Notation (json) format. At the root level of the dataset, participant demographic information, including shifted birthdates, sex, handedness at each session, shifted testing dates for the standardized assessments (using the CELF-5 testing date) at each session, and grade level (from the developmental survey) at each session are provided in the participants.tsv file. The dates were shifted by adding a random number of days ranging from 1 to 365 to the original date and then subtracting 200 years. The shifted amount was kept the same within a participant. The header variables in the participants.tsv file are further described in the accompanying data dictionary, i.e., participants.json. Psycho-educational assessment and questionnaire data as well as accompanying descriptive json files can be found in the phenotype folder, sorted by session and test. Neuroimaging data can be found in individual subject folders labeled sub- < ID > , organized by session and imaging type. Because the imaging data within a session could be scanned repeatedly and/or on different days, we added acq- < D#S# > in their file names to indicate the relative scanning day and series number. Functional magnetic resonance imaging (fMRI) task behavioral data is stored in the func folder in the appropriate ses- < sessionID > folder for each subject with a < task name > _events.tsv file and a < task name > .json file alongside their BOLD imaging data file for that same run. fMRI task behavioral data is compiled per trial and includes onset, duration, trial type, correct response, window1_response, window1_RT, window2_response, window2_RT, stim_file, calculated_response, calculated_accuracy, and calculated_RT and is stored in the < task name > _events.tsv file. fMRI task shifted acquisition date, and the converting tool that was used are also stored in the < task name > .json file. Descriptions of task instructions and event file column headers can be found at the root level of the dataset under task- < task name > _bold.json, and task- < task name > _events.json, respectively. The reconstructed scanning protocol is stored as a PDF in the derivatives folder. The E-prime programs are stored in the derivatives/Eprime folder. All stimuli files used in the functional MRI tasks and linguistic characteristics of these stimuli can be found in the stimuli folder.

## Technical Validation

All psycho-educational tests were scored twice by trained research team members and compared. In the case of a discrepancy, a third scorer would review and determine the correct score. Upon curation of the dataset, all scores were reviewed to ensure no data entry errors had occurred. All identifying information from the free response questions in the questionnaires was removed to protect the confidentiality of the participants.

Neuroimaging data was converted from standard DICOM to NIFTI format using dcm2niix version 1.0.20190902. Imaging parameters for the structural and functional images were extracted from the DICOM headers and stored in the json file alongside the imaging data. A few data files (29 functional runs and 4 anatomical runs, indicated in the readme.txt) were converted from DICOM to NIFTI using SPM8, because the original DICOM images were lost. Therefore, we manually created json files for these 33 runs. These manually created json files only contained parameters that were consistent across runs of the same type that were converted using dcm2niix. Because slice timing is not consistent across runs, we manually created the slice timing for the 29 functional runs by averaging the slice timing extracted from the other functional runs that were converted by dcm2niix of the same type. We documented these 33 runs in the readme.txt file included in the dataset.

Functional T2-weighted images were reoriented to the anterior commissure using Statistical Parametric Mapping (SPM12, https://www.fil.ion.ucl.ac.uk/spm/software/spm12/). All images were evaluated for movement due to the high likelihood of in-scanner movement in pediatric populations. Within each run, volumes that had a greater than 1.5 mm volume-to-volume motion, or global signal intensity deviation greater than 4% were marked as bad volumes using the ArtRepair toolbox 4.0^34^. The in-scanner behavioral data were converted from raw E-prime data files to text files and then information for each trial in each run was extracted for each subject and saved as tab separated values using python in the < task name > _events.tsv files. For ease of screening data for future researchers, we reported the movement evaluation as well as the averaged behavioral performance for each condition for each run at each session in tabular data files and stored that information in the derivatives/func_mv_acc_rt folder. Each tabular file (i.e., mv_acc_func_ses-5.tsv, mv_acc_func_ses-7.tsv, mv_acc_func_ses-9.tsv) includes the participant id, run name, number of bad volumes, number of chunks having more than 6 consecutive bad volumes identified using ArtRepair, shifted acquisition date, calculated accuracy, and calculated average RT of correct responses for each condition. Before calculating the averaged RT for each condition, we calculated the mean and the standard deviation of all correct trials across conditions within a run and then excluded trials with RTs that exceeded 3 standard deviations and trials with RTs that were less than 250 ms. The header variables in these tabular files are further described in the accompanying data dictionary, i.e., mv_acc_func.json. In addition, the approach used in calculating the accuracy and RT for each task is documented in the Acc_RT_Calculation.doc file within the derivatives/func_mv_acc_rt folder.

Facial features were scrubbed from all T1-weighted images by running pydeface (https://github.com/poldracklab/pydeface). Pediatric templates created using the CerebroMatic toolbox^35^ were substituted for the pydeface default template due to the young age of participants in the dataset. The default face mask was also replaced with warped face masks, which fit the pediatric templates. Visual inspection confirmed that all facial features were completely removed, and no part of the brain image was cut.

**Fig. 3:**
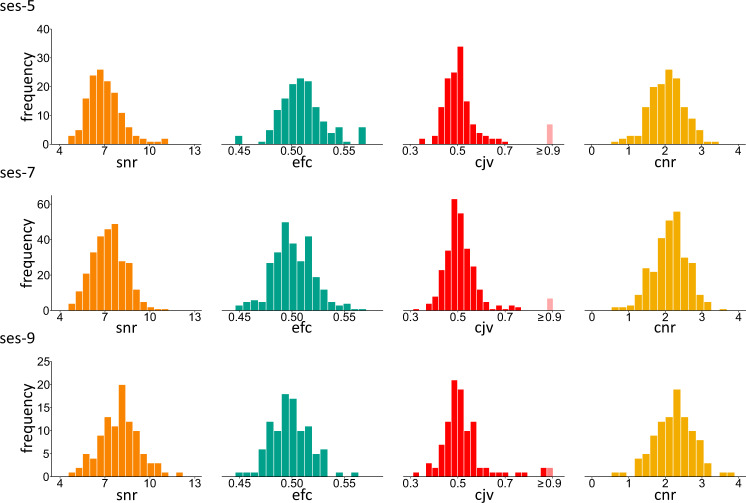
Distribution of quality metrics for the T1-weighted MPRAGE data. Distributions are based on data with the highest composite quality score per subject per session at ses-5 (n = 141), ses-7 (n = 282), and ses-9 (n = 100). Frequency represents the number of participants at a given quality score. Snr = signal-to-noise ratio, efc = entropy-focus criterion, cjv = coefficient of joint variation, and cnr = contrast-to-noise ratio. The lighter colored bar in the cjv histograms indicate extreme values.

**Fig. 4:**
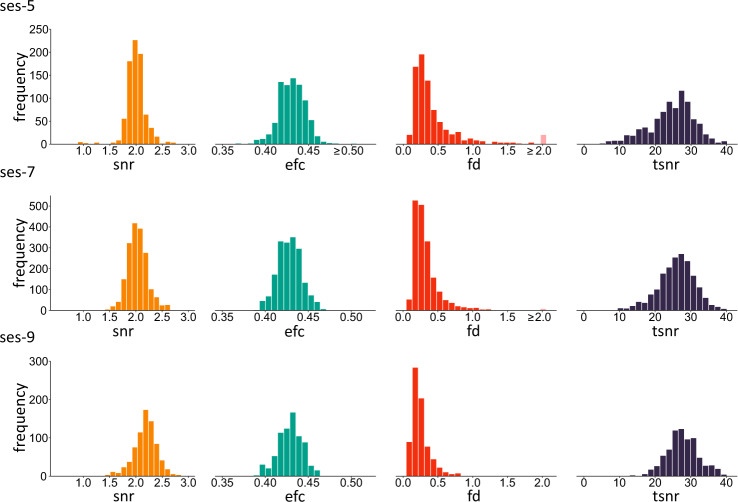
Distribution of quality metrics for T2-weighted MRI data. Distributions are based on data with the lowest framewise displacement per run per subject per session at ses-5 (n = 816), ses-7 (n = 1860), and ses-9 (n = 752). Frequency represents the number of participants at a given quality score. Snr = signal-to-noise ratio, efc = entropy-focus criterion, fd = framewise displacement, and cnr = contrast-to-noise ratio. The lighter colored bar in the fd histograms indicate extreme values.

After the removal of facial features, all T1- and T2-weighted images were reviewed with the MRI Quality Control tool (MRIQC)^36^. MRIQC PDF reports of each image are included in the derivatives/mriqc folder. Table 4 provides detailed descriptions of the selected quality metrics for the T1- and T2-weighted images from MRIQC, which are plotted in Figs. 3 and 4. Figure 3 provides histogram representations of the quality control measures for the T1-weighted images. Figure 4 shows histogram representations of the quality control measures for the T2-weighted images. Because multiple T1-weighted images were sometimes collected for a single subject at a single session, we reported a composite quality score for future researchers in the derivatives/t1_qa_composite folder to facilitate ease of screening. The composite score for each T1-weigted image at each session for each participant is stored in tabular data files (i.e., t1w_qa_ses-5.tsv, t1w_qa_ses-7.tsv, t1w_qa_ses-9.tsv). This composite score was calculated by first z-scoring the four quality measures derived from the MRIQC reports (i.e., efc, snr, cjv, cnr), reversing the negative measures (i.e., efc, cjv) and summing them together. Therefore, a higher composite score indicates a better T1-weighted image quality. Image quality metrics were slightly worse in the current dataset than those reported in previous datasets on developing children^37–39^ using MRIQC, likely because our participants were younger. Image quality metrics demonstrate a gradual improvement in data quality over development.

**Table 4:** Description of selected quality control metrics for T1- and T2-weighted images from MRIQC and selected metrics for diffusion-weighted images from FSL eddy.

Image Type	Metric	Description
T1-, T2-, and Diffusion-weighted	Signal-to-noise ratio (snr)	A measurement of quality of signal within the brain tissue. Higher values are better.
T1- and Diffusion-weighted	Contrast-to-noise ratio (cnr)	A measurement of noise indicating separation of grey and white matter tissue distributions. Higher values are better.
T1- and T2-weighted	Entropy-focus criterion (efc)	A measurement of ghosting and blurring caused by head motion. Lower values are better.
T1-weighted	Coefficient of joint variation (cjv)	A measurement of noise indicating head motion and intensity non-uniformity (INU) artifacts. Lower values are better.
T2-weighted	Mean framewise displacement (fd_mean)	A measurement of head movement across data acquisition calculated by realignment. Lower values are better.
T2-weighted	Median temporal signal-to-noise ratio (tsnr)	A measurement of quality of signal calculated as median signal over temporal standard deviation. Higher values are better.
Diffusion-weighted	Mean of absolute RMS movement (rms_abs)	A measurement of total movement in each volume as compared to the reference volume. Lower values are better.
Diffusion-weighted	Percentage of outlier slices (outlier_pct)	A measurement of data quality calculated as the percentage of outlier slices in a run. Lower values are better.

**Fig. 5:**
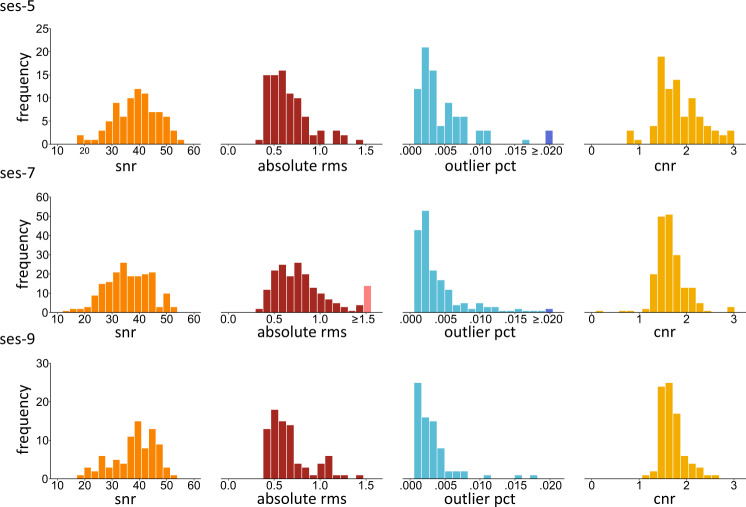
Distribution of quality metrics for diffusion-weighted data. Distributions are based on data with the lowest absolute RMS movement per subject per session at ses-5 (n = 87), ses-7 (n = 191), and ses-9 (n = 84). Frequency represents the number of participants at a given quality score. Snr = signal-to-noise ratio, rms_abs = mean of absolute RMS movement, outlier_pct = percentage of outlier slices, and cnr = contrast-to-noise ratio. The lighter colored bar in the histograms for absolute rms and the darker colored bar in the histograms for outlier pct indicate extreme values.

The quality of diffusion weighted images was assessed using the FSL eddy command (https://fsl.fmrib.ox.ac.uk/fsl/fslwiki). Movement during the scan was evaluated using the average of the absolute and relative volume-to-volume movement (RMS) derived from my_eddy_output.eddy_movement_rms and the outlier slice percentage derived from my_eddy_output.eddy_outlier_report. Signal quality of the scan was evaluated using the average of contrast-to-noise ratio (cnr) on the non-zero b-shells and the averaged signal-to-noise ratio (snr) of the b0 maps derived from my_eddy_output.eddy_cnr_maps. These indexes are reported for each run at each session in tabular data files and stored in the derivatives/dwi_mv_snr folder. Each tabular file (i.e., dwi_qa_ses-5.tsv, dwi_qa_ses-7.tsv, dwi_qa_ses-9.tsv) includes the participant id, run name, shifted acquisition date, number of volumes, mean of absolute RMS, mean of relative RMS, outlier percentage, averaged snr of b0 map, and averaged cnr for each dwi. The header variables in these tabular files are further described in the accompanying data dictionary, i.e., dwi_qa.json. Table 4 provides descriptions of the selected quality metrics for the diffusion weighted images, which are plotted in Fig. 5. Figure 5 shows the histogram representations of the quality control measures for the diffusion weighted images. Movement metrics were slightly worse than those reported in previous datasets on developing children using different quality control tools^35,37^, probably because our participants were younger. Image quality metrics of diffusion weighted imaging shows a gradual improvement as children develop. However, overall, the quality of the diffusion weighted images is good in our dataset with most data having less than 1% of outliers.

## Usage Notes

All data are publicly available under a Creative Commons CCO license. We encourage the use of this dataset for further analysis and publication under the requirement of citing this article and the dataset. This dataset was successfully analyzed using SPM for fMRI in previous publications^40–50^. The dataset is shared as a whole dataset only on OpenNeuro.org. Questions regarding this dataset can be directed to the corresponding authors or posted as a comment on the OpenNeuro.org page for the dataset.

## Data Availability

Code used to create BIDS data is located in the code directory at the root level of the dataset. The ELP_convert_to_bids.py is the main code for BIDS organization from the pre-existing file storage. It calls two sub-functions: beh_process.py, which creates events files from compiled E-prime data and dicom_proc.py, which utilizes dcm2niix to convert DICOM to NIFTI files with its corresponding json file. The ELP_acc_rt.ipynb and four excel files (i.e., Gram.xlsx, Phon.xlsx, Plaus.xlsx, and Sem.xlsx), which are located in the code/func_qa/acc_rt folder, are the code and files used to produce the calculated_response, calculated_accuracy, and calculated_RT for each trial in the events files. The code used to evaluate the movement of the T2-weighted images is located in the code/func_qa/mv folder. The main_just_for_movement.m is the main code used for movement evaluation that calls other sub-functions: realignment_byrun.m and art_global_bdl.m. SPM12 and ArtRepair need to be added before running this code. The count_repaired_rt_acc.m is the code used to count the outlier volumes as marked by ArtRepair and calculates the accuracy and RTs for each condition of each run for each task. Code used to examine the quality of diffusion weighted images is located in the code/dwi_qa folder. The dwi_eddy.sh is the main code that runs FSL eddy which calls other parameters stored in the acqparam.txt, index1.txt, index2.txt, or index3.txt files. The eddy_qa_output.sh is the code that calls the FSL eddy outputs and calculate the quality metrics stored in the derivatives/dwi_mv_snr folder. The DTI_qa_composite.m is an additional code to calculate a composite quality score for the dwi. Code used to calculate the composite score for the quality of the T1-weighted images is located in the code directory and named T1w_qa_composite.m.

## Online-only Table

**Online-only Table 1:** Standardized psycho-educational tests and subtests completed at each time point. .

Measure	Test	Subtest	Ses-5	Ses-7	Ses-9	Scores
Language Skills	Clinical Evaluation of Language Fundamentals (CELF-5)	Sentence Comprehension (SC)	*	*		RS ScS
		Linguistic Concepts (LC)	*	*		
		Word Structure (WS)	*	*		
		Word Classes (WC)	*	*	*	
		Following Directions (FD)	*	*	*	
		Formulating Sentences (FS)	*	*	*	
		Recalling Sentences (RS)	*	*	*	
		Understanding Spoken Paragraphs (USP)			*	
		Word Definitions (WD)			*	
		Sentence Assembly (SA)			*	
		Semantic Relationship (SR)			*	
		Core Language Score (CLS)	*	*	*	StS
		Receptive Language Index (RLI)	*	*	*	
		Expressive Language Index (ELI)	*	*	*	
		Language Content Index (LCI)	*	*	*	
		Language Structure Index (LSI)	*	*		
		Language Memory Index (LMI)			*	
Phonological Processing	Comprehensive Test of Phonological Processing(CTOPP-2)	Elision (EL)	*	*	*	RS ScS
		Blending Words (BW)	*	*	*	
		Sound Matching (SM)	*			
		Phoneme Isolation (PI)		*	*	
		Memory for Digits (MD)	*	*	*	
		Nonword Repetition (NR)	*	*	*	
		Rapid Digit Naming (RD)	*	*	*	
		Rapid Letter Naming (RL)	*	*	*	
		Rapid Color Naming (RC)	*			
		Rapid Object Naming (RO)	*			
		Phonological Awareness	*	*	*	StS
		Phonological Memory	*	*	*	
		Rapid Symbolic Naming	*	*	*	
		Rapid Non-Symbolic Naming	*			
Reading	Woodcock Johnson III Tests of Achievement (WJ-III)	Letter Word Identification	*	*	*	RS StS
		Passage Comprehension			*	
		Reading Fluency			*	
Intelligence	Kaufman Brief Intelligence Test (KBIT-2)	Matrices	*	*	*	RS StS
Articulation	Goldman Fristoe-2 Test of Articulation (GFTA-2)	Sounds in Words	*	*	*	RS StS
Language Variation	Diagnostic Evaluation of Language Variation (DELV)	Part 1	*	*	*	RS DELV_Status

Note: RS refers to Raw Score; TS refers to T scores; StS refers to Standardized Score; ScS refers to Scaled Score; DELV_Status is a categorical classification result based on language variation.
